# Public health practitioners as policy advocates: skills, attributes and development needs

**DOI:** 10.1093/heapro/daad102

**Published:** 2023-09-13

**Authors:** Susie Sykes, Megan Watkins, Jane Wills

**Affiliations:** School of Allied and Community Health, London South Bank University, 101 Borough Road, London SE1 0AA, UK; School of Allied and Community Health, London South Bank University, 101 Borough Road, London SE1 0AA, UK; School of Allied and Community Health, London South Bank University, 101 Borough Road, London SE1 0AA, UK

**Keywords:** health in all policy, advocacy, policy, public health competencies

## Abstract

Effecting policy change is a key strategy in tackling wider determinants of health. In England, public health sits within Local Authorities (LAs) and responsibility for ensuring health is considered across directorates increasingly falls to public health practitioners. While international professional standards expect competence in understanding policy processes, the advocacy role has been under-explored. This paper explores the professional skills, role characteristics and learning needs of practitioners advocating for the restriction of advertising high-fat, salt and sugar products in a region of England. A series of three interviews were conducted at three time points over 10 months with policy advocates leading this policy change from four LAs. Three focus groups were also held with 12 public health advocates from 10 LAs at the end of the 10-month period of data collection. Data were transcribed and analysed retroductively. Data showed that practitioners felt inexperienced as policy advocates and saw this work as different from other public health approaches. Successful advocates required interpersonal skills, knowledge of policy-making and local governance, determination, resilience, confidence, belief in their work’s value and leadership. These skills were difficult to acquire through formal education, but advocacy training, mentorship and role modelling were seen as important for professional development. To successfully implement a Health in all Policies approach and address wider determinants of health, public health practitioners need to be equipped and supported as policy advocates. The advocacy role and the complex skills required need to be more fully understood by the public health profession and prioritized within workforce development at both local and national levels.

**Contribution to Health Promotion**

- Policy change addresses wider determinants of health, crucial for improving population well-being
- The public health workforce plays an important role as policy advocates.
- This paper helps us understand the skills and development needs required in this role.

## INTRODUCTION

Responding to complex public health priorities, requires strategies to address wider determinants of health at policy level. A Health in all Policies (HiAP) (World Health Organization, 2021) approach recognizes that many policies impacting population health and health inequalities sit outside the health sector, for example in transport, education, housing and planning sectors. Municipal or local governments, with responsibilities across these sectors but with close links to local communities have been identified as the most feasible tier of government to enact HiAP (Lilly *et al.*, 2023). There is a need for public health and health promotion professionals to champion as policy advocates for health to be considered by policies across these sectors.

Advocacy is a core function of public health in promoting inclusion of health across the policy realm. Public health advocacy is defined as the ‘*combination of individual and social actions designed to gain political commitment, policy support, social acceptance and systems support for a particular health goal or programme*’ [(World Health Organization, 2021), p. 17] and advocates seek to influence policies to create circumstances that maximize the potential for community health and well-being. It is a process that occurs through media, legislative or community-based efforts and is distinct from individual health advocacy which speaks for the needs of patients or service users, because of its attention on enhancing the health of whole communities through improved policy.

Influencing policy change through advocacy can be a complex process and as Clavier and de Leeuw (Clavier and de Leeuw, 2013) point out, is not linear, involves interactions with numerous stakeholders with competing agendas and faces many challenges. These challenges are often compounded when local policy change is sought in a context linked to big national or multinational commercial interests such as in gambling, alcohol and food policy (David *et al.*, 2019, 2020). A recent review examining the factors influencing HiaP in local government, identifies policy advocates as an important but under-reported factor influencing the change process (Lilly *et al.*, 2023).

International public health competency frameworks incorporate advocacy within their standards (de Leeuw *et al.*, 2014). The Competency Framework for the Public Health Workforce in the European Region published by WHO and the Association of Schools for Public Health in the European Region alludes to advocacy skills throughout. Specifically, competence 3.4 calls for a workforce that is competent in: ‘*implementing health and social policies and plans that help to guarantee the right to equitable and effective health care and policies enabling environments favourable to health*’ [(World Health Organsiation, 2020), p. 17] and competence 7.8 ‘*advocates for healthy public policies and services that promote and protect the health and well-being of individuals and communities*’ [p. 28]. Within the UK the Public Health Knowledge and Skills Framework also calls for a workforce that can ‘*work with and through policies and strategies that improve health and reduce health inequalities and outcomes*’ (Public Health England, 2016).

While central to competency frameworks and emphasized in health promotion and public health practice (Carlisle, 2000; Gould *et al.*, 2012; Cohen and Marshall, 2017), policy change is complex. Although there are numerous policy-making and analysis frameworks, they are rarely adequately applied to public health (Howlett *et al.*, 2016; de Leeuw *et al.*, 2021). An exception is the work of Cullerton *et al.* (Cullerton *et al.*, 2016a,b,c, 2017, 2018) on public health nutrition advocacy that highlights essential advocacy skills, such as flexibility, opportunism, persistence and credibility. Concerns have been raised regarding workforce capacity, with many said to lack the necessary skills and experience for this work (Garcia *et al.*, 2015; Blenner *et al.*, 2017) hindering the potential of public health advocacy to, for example, address health inequities (Cohen and Marshall, 2017). Thus, there are calls to integrate advocacy into public health training and professional development (Blenner *et al.*, 2017). However, further exploration is needed to understand the experiences, challenges, opportunities and resources required for effective advocacy by public health professionals.

## METHODS

This study investigates the professional skills, role characteristics and professional development needs of public health practitioners in their role as policy advocates. As part of a wider realist evaluation, it examines the skills and attributes drawn on over a 10-month period by practitioners working as advocates seeking to reduce advertising of products high in fat, salt and sugar on council-owned outdoor spaces across 14 municipal areas (Local Authorities, LAs) in a region of England.

In England, public health practitioners are part of the core public health workforce in roles including health promotion, health protection and healthcare public health (United Kingdon Public Health Register., 2018). They may apply to join a voluntary register but have no common training route and come from varied backgrounds. They are distinguished from public health specialists who are formally registered and work at strategic or senior management level, having completed an accredited 5-year training program or been assessed through portfolio. Public health practitioners are typically employed within public health departments within LAs but may be employed in other settings including National Health Service, government agencies or voluntary, community and social enterprise. This paper looks specifically at those employed by LAs.

Three serial interviews were conducted over a 10-month period with practitioners from four LAs to capture reflections at different stages of the advocacy process (months 1, 5 and 10). This was important given the extended time required for policy change and to capture ‘in the moment’ rather than retrospective reflections on skills and attributes as the process progressed (Hermanowicz, 2013; Read, 2018). They were not intended to measure change over time. Three focus groups were conducted at the end of the 10-month data collection window with 12 practitioners from 10 LAs within the region. Two interview participants also attended the focus groups. Each practitioner had a similar role as a lead advocate in their own LA but differed in background and experience. They formed a Community of Improvement facilitated by two representatives of the central government Office for Health Improvement and Disparities (OHID). The interviews provided a deep examination of practitioners’ changing experience during advocacy, while the focus groups fostered idea exchange and collective reflection based on individual experiences and perceptions (Kitzinger, 1995). Interviews and focus groups were conducted online via Microsoft Teams.

Focus group and interview schedules were developed using Cullerton’s conceptual model for influencing nutrition policy within which skills required for advocacy and associated development needs are described (Cullerton *et al.*, 2018). Themes arising from an initial analysis of the interviews further informed the focus group schedule with group activities to promote discussion such as; what would you include in a job description for somebody doing this work? The research tools associated with this paper are lodged on the OSF and are available at: https://osf.io/s7bvm/. Data were analysed using a retroductive approach (The RAMESES II Project, 2017) which allows for both inductive and deductive logic as well as researcher insights to identify causes behind observed patterns. The analysts were external evaluators with a frame of reference based on experience in public health practice, education and psychology. This insight influenced the development and refining of codes and interpretation into themes. A theoretical coding framework, based on the skills identified within the conceptual model, was developed. Transcripts were organized within NVivo 12 software. To ensure familiarization, two researchers read each transcript, independently coded two transcripts using the theoretical coding framework and conducted additional inductive free-coding. Coding was compared for consistency and the framework refined. One researcher completed subsequent coding, while the second coded a random 20% sample and ambiguous data. Regular discussions were held between the researchers. Themes were identified through analysis and reflection allowing relationships between themes to be determined.

## RESULTS

Seven themes were identified that represent skills, attributes and professional development needs that public health advocates felt were important: politically astute interpersonal skills, policy subject and process expertise, determination and resilience, autonomy, integrity, organizational and professional permission for advocacy and learning to be an advocate.

### Politically astute interpersonal skills

This theme captured the complex interpersonal skill-set informed by political insight required by a policy advocate including: the ability to navigate complex relationships and power dynamics, understand and manage internal politics, build and manage effective relationships, influence and build support for policy positions, communicate effectively and distil complex information for diverse audiences.

Being able to collaborate with others in complex relationships and build and manage effective relationships, often with people outside of public health networks and beyond usual working contacts, was identified as important. Participants demonstrated how they were able to do this on both a formal and informal basis, building on previous interactions and on an ongoing basis.

Participant E: My successes are down to the fact that I’ve taken time to build a relationship with someone, and that relationship sometimes might be based on really informal chit chat, not even work-related sometimes, so that you develop a relationship with someone based on mutual respect, and it takes time to do that, but without that I absolutely would have failed at so many things that relied on someone else’s goodwill to make it happen and they’ll kind of do it as a favour to you because they like you and they know that you’ll reciprocate it.

Participant F: I can only build on what xxx said… so those things are really important in this role and other roles having personal relationships with people to progress things and having good working relationships is really important, it always has been (Focus group 2)

The key purpose within these relationships was to influence and build support for the policy position with the quote below demonstrating the importance of making persuasive, resonating arguments:

It is all about talking to one degree or another because at the end of the day to advocate is to try and influence and change people’s opinions, and you’re only going to do that through talking to them. You can talk with facts or whatever, but it’s about making a coherent argument and bringing people along with you. (Focus group 2)

Diplomacy skills were crucial for exerting influence, especially when collaborating with senior members of the organization, individuals with strong personalities, and those who hold ideologically driven positions that may not align with this particular, politically sensitive policy change. These are likely to have been particularly important given the need to work across levels of seniority and also with both elected members and employed officials.

Participant E: I remember when I first approached a colleague in the council that I needed to speak to, to get their support to agree to take this work forward, but she’s renowned for being quite difficult, so I was quite nervous beforehand and it turned out much better than I thought and I did draw on every ounce of diplomatic skills that I’ve got in order to pitch it right in a way that I knew or I suspected she’d warm to, and fortunately she did.

Participant G: I’d completely agree with xxx, they are all absolutely key skills!… you’ve had to be quite good at having those interpersonal conversations, the influencing and the distilling down of the information, to just get things moving along (Focus group 2)

The distilling of information mentioned in the above exchange and communicating complex and technical information in an understandable, persuasive way for different audiences in order to ‘take people with you’ was frequently referred to, with the example below demonstrating the challenge of speaking the different ‘languages’ required in different departments:

And, actually, if we’re really trying to make a policy change … and we’re trying to reduce health inequalities and we’re trying to bring people on board, we really need someone that can talk to different levels…., there’s certain areas where I’m just like, ‘I don’t know how to explain this properly.’ Like I’m really struggling to get my language right to talk to. The one that comes most recently is talking to people in Education about health. That is horrendous! I’m just stuck there, thinking, ‘I don’t know how else I can say this.’ So, I think trying to get, for me, someone who can really speak at different levels is really, really important because the way you would talk to a councillor and get them on board is so different. I think that’s a real challenge and real skill. (Focus group 3)

Managing these conversations requires political astuteness and understanding of: people’s position, how receptive they might be, what power they have and that they may be operating within competing agendas:

Participant C: the most challenging thing about this has been the internal politics and knowing who to speak to and who not to speak to and when to speak to people, so a really strong political – with a small p and a big P, awareness is really crucial, being able to understand the nuances of what’s going on with relationships between other people, who is friendly with who…. So probably someone who knows the system already quite well is really important.

Participant D: Yes, I think it’s someone who understands the politics and the relationships and the personalities that are there. (Focus group 1)

The skills reflected in this theme enable policy advocates to communicate effectively, build relationships, negotiate and influence others in a politically sensitive manner to what some see as a politically or commercially contentious policy issue, allowing the policy advocate to navigate complex political environments, build coalitions and build political will.

### Policy subject and policy process expertise

This theme outlines the importance of having subject-area knowledge of the policy-making process to complement politically astute interpersonal skills. Participants emphasized the need for a detailed knowledge of the policy subject and its supporting evidence base, as well as the ability to use this knowledge to strengthen arguments. The examples show the importance of using this knowledge to influence the relationship-building skills described in Politically astute interpersonal skills.

The need for detailed knowledge of the policy subject and its supporting evidence base was seen as imperative:

Somebody who’s well versed and knowledgeable of the area, of the policy. (Focus group 1)

Participant I: Yes, I would agree with xxx again,…. all of our work’s really strengthened with evidence base. So having that knowledge and using the evidence that you have really helps.

Participant J: I agree. Certainly around evidence, definitely. (Focus group 3)

Given the frequently expressed concerns about financial and commercial implications of this particular policy change, knowledge of health economics to support the case was identified as desirable:

There is this bit of work around health economics and how you make the argument in terms of money…. And I think that’s, for me, strengthened the argument. (Focus group 3)

Knowledge of the policy area needs to be complemented with knowledge of the policy-making process, both theoretically and as it is practiced within their local jurisdiction. The difference between theory and practice and how this also varies from area to area is captured in the data below:

I think there’s something about understanding the policy process as well from how you get from A with nothing to B with the policy adopted, so I’d probably want somebody who had more experience in going through that process and have that understanding around all the governance and all the democratic services stuff. (Focus group 1)

Every local authority having different ways of working and different things, so for us as practitioners is that not using our intelligence and knowledge of our own local authorities to decide who we do need to talk to and who we can leave for a bit. (Focus group 1)

This combination of both theoretical and technical knowledge was seen as difficult to acquire but essential in understanding how to move the advocacy process forward and identify opportunities for action.

### Determination and resilience

Progressing policy change was seen as slow and complex requiring positive attitudes, determination, resilience and patience as these personal reflections demonstrate:

Participant B: I don’t know if they’re skills or traits, but a lot of patience, resilience, determination and persistence….

Participant C: …Yes, and I think it’s understanding and having that knowledge when you go into it and not being disheartened, that it is going to take a long time, and that is just normal, so that’s one of the benefits from it. (Focus group 1)

It’s the ability to keep picking yourself off the floor, brushing yourself down and saying what should we try now. You’re in it for the long game, things don’t happen overnight and usually every success will have come from lots of failure. (Focus group 2)

This resilience was more likely to be achieved where a belief and passion in the policy and its potential long-term contribution to health outcomes were held:

Participant A: It’s the quote about the oak tree, that society grows great when all men plant the trees that they will never see, and the fact that you were willing to put so much effort into something and you might never see it, it might be the children that are growing up today, you might make a change and then 20 years from now as young adults they’re living in a much better space …. But then you might be gone, you might be retired or off doing something else, but if you’re willing to do it.

Participant B: … So it’s a bit of a thankless task, but it’s not if you take from it that you know that you’re trying to make people’s lives better then there you go, if that’s enough for you then that’s enough for you. (Focus group 2)

This determination and resilience is likely to be important in fostering the complex relationship building described in Politically astute interpersonal skills.

### Autonomy

Participants were often the only member of their team with a defined responsibility for policy change in this area. The ability to work autonomously, drawing on leadership skills to keep the issue on other people’s agenda was important:

Someone who is quite self-directed is often quite useful in terms of not only have they got the drive to take ownership of something, but they’re happy as well to manage it in their own way… if you’re really confident enough to progress that yourself without going back every time to whoever would be the person you would be reporting to on that. (Focus group 2)

This ability to work autonomously is likely to be important given the complex and extended time that policy change takes and will influence the policy advocate’s ability to enact the complex interpersonal skills described in Politically astute interpersonal skills. It is a characteristic that can be seen to be applied to both the individual and to the role.

### Integrity

Participants felt that the way they were perceived by local stakeholders was important and that they needed to be respected and credible. Similar to the ability to work autonomously, integrity was seen as important for both the individual and integral to the role:

Participant C: I can see the credibility that has come with public health as a result of the response to Covid has massively helped. Before Covid you would be hard pushed to find anyone in a LA who really knew what public health did and valued their expertise, their honesty, their impartiality and the following of evidence and intelligence, and now I think we’ve seen a shift in that. So, I think with public health comes a massive amount of credibility.

Participant D: …: I was just going to add to what xxx said, I think it comes back to what we’ve been discussing before about credibility, someone that’s quite well respected or has shown that they’ve got the knowledge and they’ve got the skills and they’ve built those relationships up to be quite well respected, and therefore when the time comes when you’ve got that big ask of them, they are more willing to do that. (Focus group 1)

Trust and credibility were also seen as important at a professional level. Understanding of public health was seen to have increased since the pandemic, enhancing credibility and visibility of the profession, providing an important platform for advocacy:

I think Public Health have got very well-established in there and people know what we do now and I also think, partly to do with Covid, there’s a slightly different respect there. (Interview, participant 2)

You know, our Director, he’s very well respected. I think now, even particularly after COVID and the handling of COVID locally, again we’ve gained a lot of …I guess it’s just our … I don’t want to say ‘leverage’, but I think people understand Public Health more now. (Interview participant 4)

### Organizational and professional permission to be an advocate

A further theme was a need to have ‘permission’ to act as a policy advocate. Participants felt strongly that advocacy work represented a different way of working to other strategies within public health, such as project implementation and commissioning, which were seen to have clearer processes and guidelines for delivery:

Participant I: And service work: it feels it’s very practical. You’ve got a service specification; you’ve got procurement; you can do all the performance management. For me, I’ve got more skills in that and can see the process of that. It doesn’t feel as straightforward in terms of the policy work…And I’ve never felt so conscious of the sensitivities around doing something like that that this brings. So, it does feel quite new, and, for me, it’s definitely slowed the process down.

Participant J: Yes, I was going to agree with xxx… I find that, yes, the process of that commissioning that we do is a lot more simple… councillors – they want to see something tangible… They want to see something that they can actually touch and feel. So a policy change: you don’t really touch and feel that particularly. And a lot of people, unless they’ve been really involved it, won’t even notice it. (Focus group 3)

The nebulous nature of advocacy meant that it was often squeezed into an existing workload without clarity of objectives or identified milestones. The need for a named and continuous policy advocate was crucial but they needed to be properly resourced and supported:

I suppose at the very beginning, you know, laying out the capacity requirements for this would have been useful, so that we can have a better understanding of what the demands are in terms of our capacity. I suppose a ballpark guesstimate…, at the very beginning in terms of the amount of time that needs to be spent on it, because that then helps you to justify that time to your superiors. (Interview: participant 3)

And it’s continuity, isn’t it, like you say, if there’s a political change or there’s a change within our team, you know, it’s making sure that there’s some continuity to this work, because if it gets started and then halted, you know, reputationally as well if we’re engaging with people, the actual investment in this has to have some continuity. (Interview, participant 4)

Some participants also felt that clarity was needed about the permission for advocacy to take place within an organization. As practitioners working within a municipal local government body, the mandate for them to operate as political advocates was unclear and, in some cases, uncomfortable. Being seen to question the position of the elected representatives and suggesting a different policy position was challenging:

But, on a local scale, I think there’s more challenges to that because of the power we hold as individuals – health improvement team members – and whether or not it fits with the views of the organisation. And I think, if I’m honest, in some ways, some officers – and I’d probably put myself in that pool – can be a little bit apprehensive to really push their advocacy role because you don’t want to be seen as the person who’s constantly trying to challenge things – a bit like a dog with a bone – trying to push these things through. And I think, certainly for our authority, there’s work to be done about ‘What permissions do we have for advocacy?’ And that sounds like quite a strong word, but I suppose that’s what it boils down to. (Focus group 3)

I’ve never felt as if I was able to go out and rally the troops with a view to making a change internally because there’s always a party line to toe. (Focus group 3)

This lack of clarity about the organizational permission to act as a policy advocate meant practitioners often felt they did not hold enough power to effect change or access to the spaces where decisions are made or if they veered too far into the role of activist, they might jeopardize their influence:

You’ve got to be in the room to change anything, but she’s no longer in the room because they’ve stopped inviting her. So, she is frustrated, she’s not really achieving what she wants to achieve. (Focus group 2)

### Learning to be a policy advocate

Many of the skills, particularly interpersonal skills, that were seen as underpinning the role of the advocate were often described as innate character traits and difficult to acquire through formal training:

Yes, I’ve got to be honest, I do think that these skills you’ve either got or you haven’t But ultimately if you haven’t got those skills or the basics of them, I personally don’t think you can train it. (Focus group 2)

For many, the development of those skills could only come through experience, which many felt they did not have. Advocacy had not been an area in which participants had worked previously and they did not feel well prepared:

I think experience gives us what we need, and my approach must be different to what it was when I was in my twenties, you learn so much in this job over the years. (Focus group 2)

So, yes, this is a new role for people but, actually, I’m not sure that anyone’s really got the skills to do this from a standing start. (Participant 4)

While formal training was not prioritized, learning from senior colleagues and those with knowledge and expertise in policy change were seen as valuable resources that participants relied on. It was felt that development could happen through role modelling and learning by example, enabling good habits to be picked up:

If you place somebody with somebody that is really good at relationships, and even if they’re not necessarily that good at it themselves, they watch and they learn, and they do look at you and think she managed to achieve something there that I’d have thought was impossible and she did that by doing so and so, I’m going to remember that. So, they do pick up good habits in that way, and the opposite also happens. (Focus group 2)

As well as informal development, more formalized opportunities for mentoring and a practical steer from senior colleagues with the subject expertise and experience were valued:

And having that guidance and support that I would get at the consultant level is really important to me to give me that technical support and their experience and utilise that. Because, to embark on a conversation with members at that real high level of local government process is quite daunting. And I think, having that – for me – consultant level support to steer through the process is really valuable. (Focus group 3)

So having colleagues that have got that experience is really helpful in developing your own approach as well as tailoring it to your own area, if you’ve got people who have done similar work in terms of influencing policy in the past and they know people as individuals they can give you the sort of benefit of their expertise. (Focus group 2)

Participants felt that while public health specialists received training around policy development during their training programme, this was not something that practitioners had much access to. Whilst acknowledging that some advocacy skills could not be developed through training, understanding processes of policy change would be useful. Training tailored to specific local governance contexts or delivered by those who had achieved a similar policy change was desired:

I think for me it probably would have to be internal training on the processes and policies that are in place, and how you get from A to B. (Focus group 1)

These findings suggest that while some aspects of advocacy are difficult to teach, support and guidance from experienced colleagues, as well as targeted training, could assist practitioners in developing the necessary skills for effective policy advocacy.

### Thematic map

**Fig. 1:**
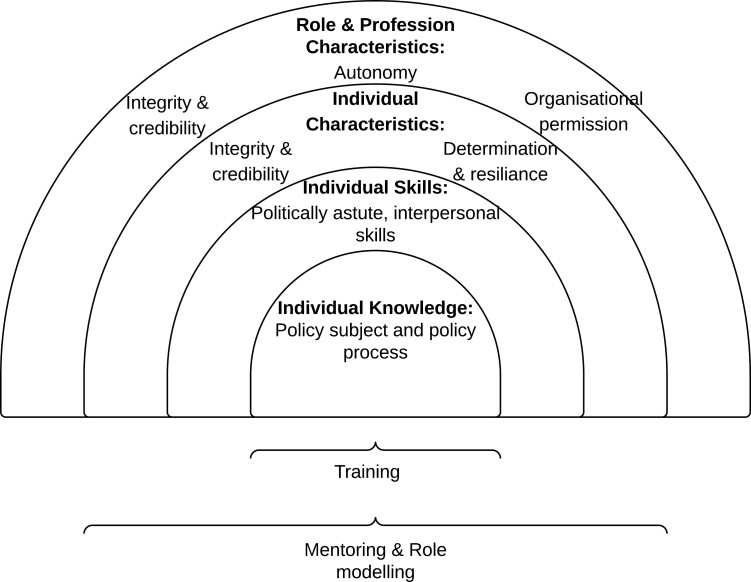
Thematic map of findings.

The themes identified in the findings demonstrate the professional skills, role characteristics and professional development needs of public health practitioners in their role as policy advocates. They can be seen to exist at the individual level but also relate to the role itself and to the profession. Knowledge is important in supporting the enactment of skills while the characteristics at an individual, role or profession level are important in fostering them. The relationship between these is captured in Figure 1.

## DISCUSSION

Influencing policy to ensure health is considered across the policy realm is fundamental in addressing the wider determinants of health and reducing health inequalities (World Health Organization, 2018) with public health practitioners well positioned for this role (Cullerton *et al.*, 2016b). However, many practitioners engaged in advocacy lack experience and feel ill-equipped (Cohen and Marshall, 2017). This study is important because it helps elucidate the skills, attributes and support required for successful advocacy. While this study has focussed on the UK practitioner role and policy changes relating to the advertising of unhealthy products, the skills identified are relevant to all those in the public health profession and to typical public health work addressing priorities such as tobacco control and gambling related harms. The results do show that this area of policy change is politically, ideologically and commercially sensitive and so the need for political adeptness and astuteness were particularly important. However, while issues and contexts may vary, and some policy changes may be more contentious and complex than others, there is transferability of advocacy processes as well as the skills and learning needs identified in this study (Eversley and Pitt, 2022). The findings offer insight into workforce requirements in different policy change contexts, facilitating appropriate professional development and support for the public health community.

Despite the recognition in public health competency frameworks of understanding and influencing policy processes, the barriers that practitioners face in health advocacy due to a lack of skills, knowledge, confidence and experience are quite well documented. (Cullerton *et al.*, 2016b; Mahas *et al.*, 2016; Kerr *et al.*, 2017; Lewis, 2020). Internationally, advocacy has been identified as a gap in public health training curricula (Hines and Jernigan, 2012; Blenner *et al.*, 2017) and concerns have also been raised that in the UK even those reaching the end of Public Health Speciality training as public health registrars have not been taught the advocacy skills they require (Murray and Leigh-hunt, 2019) and that further investment in the development of policy advocates is required (Cullerton *et al.*, 2016b) at both practitioner and speciality levels. Hines and Jernigan have identified two specific challenges in incorporating advocacy into curricula (Hines and Jernigan, 2012). The first challenge is the skills of academic staff to effectively teach advocacy, while the second challenge lies in the undertheorized and under-evaluated nature of advocacy work. This lack of evidence-based content makes it difficult to develop curricula that are relevant and transferable. Furthermore, unanswered questions remain about which theories and skills should be taught in public health advocacy curricula. Hancock expands on these challenges by suggesting that advocacy is often absent from public health curricula due to its perceived unpopularity and being seen as ‘unprofessional’ and ‘unscientific’ (Hancock, 2015).

Notwithstanding the view that theoretical and technical knowledge of the policy-making process should be included in training curricula, participants in this study argued that specific advocacy skills and locally specific political understanding were difficult to acquire through formal training routes. This is perhaps another reason why advocacy is not fully embedded within public health education and professional development. The findings that role modelling and mentoring are important strategies for skills development in this area are supported by Murray and Leigh-hunt (Murray and Leigh-hunt, 2019) who advise that in the absence of formal training, practitioners should identify a role model or mentor with proven influencing skills, even in the face of opposition. They also advise that advocates form coalitions of like-minded people with shared objectives, suggesting the voluntary, community and social enterprise sector might be well placed to provide these. Their other suggested strategies for successful advocacy preparation include having an in-depth knowledge of the evidence base, an ability to communicate a compelling narrative and courage, and as such they echo the findings of this study. The high value placed on localized knowledge and informal training suggests that public health specialists with experience of advocacy should be identified locally to provide organized mentorship to practitioners in navigating policy systems and processes. The creation of communities of practice would help advocates problem-solve and share practice reflections.

The legitimacy of advocacy work may be hindered by the acceptance of employing organizations. Possibly opposing elected bodies and the lack of a clear delineation of political involvement in an individual’s role are clear challenges (Carlisle, 2000). Such perceptions are reflected in titles of papers drawn on here including ‘Training to be unpopular’ (Murray and Leigh-hunt, 2019) and ‘Advocacy: it’s not a dirty word, it’s a duty’ (Hancock, 2015). In the England, the transfer of the public health function from the NHS to LA structures aimed to bring it closer to policy-makers responsible for determinants of health (Department of Health, 2011). However, our findings suggest that positioning within a political organization may lead to an expectation that public health delivers policies in an apolitical manner aligned with the elected councillors’ manifesto. The lack of independence of public health bodies from government entities has been identified as a barrier to advocacy (Cohen and Marshall, 2017). While fears about being overly political may be unfounded, it has been argued that public health has a duty to raise a ‘call to arms’ (Kerr *et al.*, 2017; Demaio and Marshall, 2018). The intrinsically political nature of public health has been well established (Greer *et al.*, 2017; Coughlan *et al.*, 2021) and the concept of HiaP has gained recognition from LA supporting bodies (Local Government Association, 2016).There is a need for public health to redefine its advocacy function or, as Carlisle describes it, ‘self-advocate’ for the legitimacy of the advocacy role (Carlisle, 2000) and clarify the expectations of employing organizations regarding advocacy for professionals in this field. Additionally, better understanding of the advocacy role is required within the governance structures in which public health professionals operate.

These findings also highlight the importance of policy advocates being perceived as credible and trustworthy. This aligns with Carlisle’s conceptual framework for health promotion advocacy (Carlisle, 2000) which emphasizes the significance of trust, credibility and perceived expert status in representational and prescriptive advocacy focussed on legislative action for populations, as is the case in this example of advocacy, rather than in more egalitarian or community led advocacy. A study by Geiger on the credibility and persuasiveness of public health advocates before the pandemic showed that while they were recognized as experts, they were not considered more trustworthy or persuasive than ‘non-experts’ (2022). By contrast, studies in the UK during the COVID pandemic indicated high levels of trust in public health professionals by policy-makers, especially in the early stages of the pandemic (Cairney and Wellstead, 2020). This is reflected in findings here that local advocates felt the pandemic provided opportunities for increased visibility and stronger relationships between public health and policy-makers across different policy areas. Nevertheless, trust between advocates and policy-makers is dynamic and fragile and findings from this study illustrate the importance of advocates being seen as both credible and as having integrity. More needs to be done to understand how relationships of trust can be built and maintained in this specific policy (Cvitanovic *et al.*, 2021).

## LIMITATIONS

This study was conducted in LAs across one region in England, and while different issues might apply in countries with different structures and governance contexts, the variety of governance structures in this region did not appear to influence experience. The study focussed on one specific policy change, limiting the transferability of findings to other areas. It also focussed on public health practitioners rather than the entire profession. Only four participants were included in the longitudinal interviews instead of representing all LA areas. However, the analysis confirmed the consistency between focus group and interview data. The study did not include data from wider stakeholders or OHID representatives, which could have provided additional insights.

## CONCLUSIONS

Public health practitioners acknowledge the importance of policy change for population health but feel lacking in experience, training and readiness for advocacy. Advocacy is seen as distinct from other public health strategies, requiring complex skills and knowledge. Formal training should include a stronger focus on policy advocacy, complemented by locally developed informal opportunities to navigate local processes and develop interpersonal skills. The public health profession should advocate for greater understanding, acceptance and trust in the advocacy role among decision-makers and practitioners.
